# Quantum-Chemistry Study of the Hydrolysis Reaction Profile in Borate Networks: A Benchmark

**DOI:** 10.3390/molecules29061227

**Published:** 2024-03-09

**Authors:** Francesco Muniz-Miranda, Leonardo Occhi, Francesco Fontanive, Maria Cristina Menziani, Alfonso Pedone

**Affiliations:** Dipartimento di Scienze Chimiche e Geologiche (DSCG), Università degli Studi di Modena e Reggio-Emilia (UNIMORE), Via G. Campi 103, 41125 Modena, Italy; francesco.munizmiranda@unimore.it (F.M.-M.); 228357@studenti.unimore.it (L.O.); francesco.fontanive@univr.it (F.F.); mariacristina.menziani@unimore.it (M.C.M.)

**Keywords:** DFT, MP2, glasses, borate, hydrolysis

## Abstract

This investigation involved an ab initio and Density Functional Theory (DFT) analysis of the hydrolysis mechanism and energetics in a borate network. The focus was on understanding how water molecules interact with and disrupt the borate network, an area where the experimental data are scarce and unreliable. The modeled system consisted of two boron atoms, bridging oxygen atoms, and varying numbers of water molecules. This setup allows for an exploration of hydrolysis under different environmental conditions, including the presence of OH^−^ or H^+^ ions to simulate basic or acidic environments, respectively. Our investigation utilized both ab initio calculations at the MP2 and CCSD(T) levels and DFT with a range of exchange–correlation functionals. The findings indicate that the borate network is significantly more susceptible to hydrolysis in a basic environment, with respect to an acidic or to a neutral pH setting. The inclusion of explicit water molecules in the calculations can significantly affect the results, depending on the nature of the transition state. In fact, some transition states exhibited closed-ring configurations involving water and the boron–oxygen–boron network; in these cases, there were indeed more water molecules corresponding to lower energy barriers for the reaction, suggesting a crucial role of water in stabilizing the transition states. This study provides valuable insights into the hydrolysis process of borate networks, offering a detailed comparison between different computational approaches. The results demonstrate that the functionals B3LYP, PBE0, and wB97Xd closely approximated the reference MP2 and CCSD(T) calculated reaction pathways, both qualitatively in terms of the mechanism, and quantitatively in terms of the differences in the reaction barriers within the 0.1–0.2 eV interval for the most plausible reaction pathways. In addition, CAM-B3LYP also yielded acceptable results in all cases except for the most complicated pathway. These findings are useful for guiding further computational studies, including those employing machine learning approaches, and experimental investigations requiring accurate reference data for hydrolysis reactions in borate networks.

## 1. Introduction

Borate networks, which are particularly prominent in materials such as boron–silica glasses, are crucial in various applications, ranging from consumer products like Pyrex to advanced industrial materials. These networks’ significance stems not only from their widespread usage but also from their unique chemical and physical properties, making them subjects of extensive research. In particular, borate and borosilicate glasses have been shown to be able to promote the regeneration of enamel, bone, and nervous tissues when in contact with physiological fluids [1,2,3]. To enhance these functions, the dissolution rate of the glass in a water solution must match that of growth of the replaced human tissues. Unfortunately, the development and large-scale use of these glasses is hindered by a lack of knowledge of their chemical durability in physiologic conditions. This knowledge gap stems from the fact that a vast majority of investigative efforts in the field of glass corrosion has focused on silicate glasses and their derivatives, but the principles of silicate glass corrosion (e.g., composition–reactivity correlations) generally do not apply to borate glasses [4,5].

The hydrolysis of borate networks is a fundamental aspect of their chemistry, and knowledge of this process is essential for applications and a theoretical understanding. However, experimental studies in this area face significant challenges due to the complexity of the reactions and the difficulty in accurately measuring the reaction pathways and energetics. G. Werding and W. Schreyer [6] provided valuable insights into the behavior of borosilicates and selected borates under various conditions. The interaction between boric acid molecules/metaborate ions and water molecules was studied by Zhu et al. [7].

In addition to the challenges in studying borate hydrolysis, there was significant research in related areas of boron chemistry, which offers insights into the broader context of borate interactions. For example, studies on boronic acids and their derivatives provided an essential understanding of boron-based molecular recognition and sensor applications. The versatility of boronic acids in sensing and imaging applications was demonstrated by Nishiyabu et al. [8], Dai et al. [9], and Sun et al. [10]. Chan et al. [11] and Lippert et al. [12] have also contributed to the understanding of boronic acid chemistry, particularly in the context of bio-orthogonal reactions and fluorescence imaging.

Furthermore, the saccharide recognition capabilities of boronic acids, as explored by James et al. [13,14], provide a framework for understanding the interactions between borate networks and other molecular systems. The work of Jin et al. [15] on carbohydrate recognition by boronolectins and lectins illustrated the potential of borate and boronic acid systems in biological contexts.

Thus, while the experimental study of borate hydrolysis remains a challenging field, the broader research on boron chemistry, including boronic acids and their derivatives, has provided valuable insights. These studies not only enhanced our theoretical understanding but also had significant implications for various practical applications, from sensor development to biological recognition. As the field continues to evolve, the integration of experimental and computational approaches will undoubtedly yield a more comprehensive understanding of borate chemistry.

Indeed, the hydrolysis mechanism of borate networks is crucial for understanding the chemistry of materials like borosilicate glasses. Zhou et al. [16] delved into the specifics of pentaborate anion hydrolysis, shedding light on the intricate dynamics of borate networks in hydrolytic conditions, and suggested that the formation of ring structures plays a role, with hydrolization occurring at the bridge atom.

An in-depth look into the molecular events at the borosilicate glass–water interface, which is fundamental for understanding the interactions of borate networks with water, was achieved by Jabraoui et al. [17] using non-hybrid exchange–correlation functionals; they showed that hydrogen bonds between water and the oxygen atoms of a glassy borosilicate network play a role in the binding of the water to the glass.

Kagan et al. [18] contributed to the understanding of the dissolution processes in silicate networks, which is particularly relevant for the study of materials similar to borate networks, and found activation energies within the 15–40 kcal/mol with classical potentials.

To expand the application of borate networks, we need to develop a rigorous atomic-level fundamental understanding of the mechanisms and kinetics of the dissolution of these systems in water. Particular emphasis and attention should be given to explore the (i) atomistic origins of chemical and structural drivers that control the dissolution kinetics of borate networks and (ii) underlying mechanisms that govern glass dissolution.

Although a few systematic and important benchmark studies for hydrolysis reactions exist, these have been carried for organic reactions and their extension to borate networks is not obvious [19,20].

Therefore, in this study, we focused on the process of hydrolysis of a boronic dimer in three different pH conditions, namely neutral, acidic, and basic, with a certain number of explicitly treated water molecules, to extract the reaction pathways and energies. This is a first benchmarking study of DFT functionals against the MP2 and CCSD(T) calculations needed to individuate a low-cost level of theory to investigate more complex and large systems as well as to produce quantum mechanical databases for the development of reactive force fields and/or Machine Learning Potentials and to feed kinetic Monte Carlo Models to study borate and borosilicate glass dissolution at larger space and time scales.

## 2. Results and Discussion

### 2.1. Models

**Figure 1:**
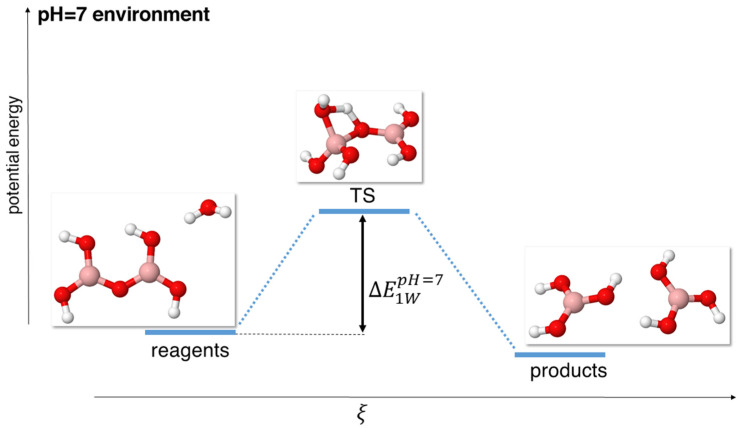
Scheme of reaction pathway for the hydrolysis mechanism at neutral pH with a single water molecule. ξ represents the reaction coordinate. The energies are not to scale; see Table 1 for a complete report on the energy values.

To model the hydrolysis reaction, we have considered a small network formed by two B atoms, connected by a bridging oxygen atom as the reagent (see reagent in Figure 1). The overall starting coordination number of each boron atom is three, and the non-bridging oxygen atoms are saturated with hydrogens to complete their coordination and to produce a neutral compound. We carried out attempts to simulate it without saturating the compound with hydrogens but the excessive negative charge on oxygen atoms led to unrealistic proton transfer from the water molecules. The products of the hydrolysis reaction were, instead, two B(OH)*_n_* compounds (with *n* equal to either 3 or 4).

In addition to these compounds, we introduced explicit water molecules to model a pH 7 environment, and H_3_O^+^ and OH^−^ ions to model acidic and basic conditions. For the neutral, acidic, and basic hydrolysis pathways, we also considered systems with two additional water molecules, as they are enough to create a ring-like transition state that can alter the energy barriers.

All the possible reactions pathways involved at least one transition state with a tetra-coordinated boron atom.

### 2.2. Assessment of the Basis Set and Ab Initio Levels of Theory

We considered the stationary points of the reaction pathway connecting a B-O-B containing unit, namely the B_2_O_5_H_4_ compound, and two separated orthoboric acid molecules, in an environment without a single water molecule, as a reference to check the reliability of the basis set employed as well as the MP2 level of theory. In fact, this was the simplest model studied here. The geometry of the stationary point is illustrated in Figure 1 and the model itself is discussed in more detail in Section 2.3.

The stationary point was first found at the MP2/6-311++G(d,p) level of theory combined with PCM. 

We performed single-point CCSD(T) [21]/Apr-cc-pVTZ calculations on each of these geometries, combined with PCM, to check that the MP2-computed energy barriers were reliable in comparison with the CCSD(T) level of theory. This was performed as Gaussian 16 currently does not allow geometry optimization at the CCSD(T) level of theory.

The computed energy differences are reported in Table 1, and suggest that CCSD(T) single points reproduce the results of MP2 optimizations with errors < 0.01 eV for the transition state barrier, and just slightly larger for the energy of the products.

**Table 1:** Energies for the stationary points of the reaction pathway at neutral pH with 1 water molecule computed with MP2 and CCSD(T) methods and different basis sets. The energies have been rescaled to have the energy of the reagents put to zero. The energy of the TS coincides with $\Delta E_{1W}^{pH=7}$.

Level of Theory	Stationary Point	Energy/eV
Optimized MP2/6-311++G(d,p) + PCM	Reagents	0
	TS	2.030
	Product	−0.015
Single-point CCSD(T)/Apr-cc-pVTZ + PCM	Reagents	0
	TS	2.038
	Products	−0.026
Optimized MP2/6-311++G(2d,2p) + PCM	Reagents	0
	TS	2.026
	Products	−0.022
Optimized MP2/6-31+G(d) + PCM	Reagents	0
	TS	2.042
	Products	−0.034
Optimized MP2/6-21G + PCM	Reagents	0
	TS	2.373
	Products	−0.009
Optimized MP2/LanL2DZ + PCM	Reagents	0
	TS	2.118
	Products	−0.011

Also, we checked the effect of the basis set, in particular, using the 6-21G, 6-31+G*, 6-311++G(d,p), and 6-311++G(2d,2p) basis sets and the combined basis-set-pseudopotential LanL2DZ [22], optimizing the stationary points at the MP2 + PCM level of theory, as also reported in Table 1. 

The energy differences remained well below the 0.01 eV threshold for the 6-311++G(d,p) and 6-311++G(2d,2p) cases, and the B-O_bridge_ distances of the transition state changed from 1.613 Å (tetrahedral B) and 1.414 to 1.616 Å (tetrahedral B) and 1.399 Å, respectively. Overall, expanding the basis set seemed to only marginally affect the energies and very negligibly affect the structure of the models we adopted here. On the contrary, using smaller basis sets led to increased differences in the energy barrier, which were particularly evident in the case of the 6-21G basis set (>0.1 eV).

Overall, we considered the 6-311++G** as a good compromise between accuracy, computational burden, and availability in the many possible quantum chemistry codes.

### 2.3. Neutral pH System

To study the hydrolysis mechanism in a neutral aqueous environment, we found and considered two possible different pathways, one including a single water molecule (1 W) and another one (3 W) exploiting the presence of three water molecules.

The structures associated with the pathway 1 W are reported in Figure 1 of Section 2.2, as we used this model (the simplest one) to also assess the validity of the ab initio and basis set levels of theory.

Following pathway 1 W, we found a transition state (TS) resulting from the nucleophilic attack of the O atom of water on a boron atom, and at the same time, the formation of a bond between a hydrogen atom of water and the bridging oxygen atom of the B-O-B network. This mechanism thus resulted in a very unstable TS (2.03 eV at the MP2 level of theory) constituted by a 4-term ring (B-O_water_-H_water_-O_bridge_). Indeed, the $\Delta E_{1W}^{pH=7}$ energies, i.e., the difference between the computed energy of the transition state and the energy of the reagents (see Figure 1), were the highest that we found for any mechanism, confirming the high unlikely and unstable nature of this TS and, consequently, of this reaction pathway. The actual energies calculated with different functionals are reported in Table 2.

**Figure 2:**
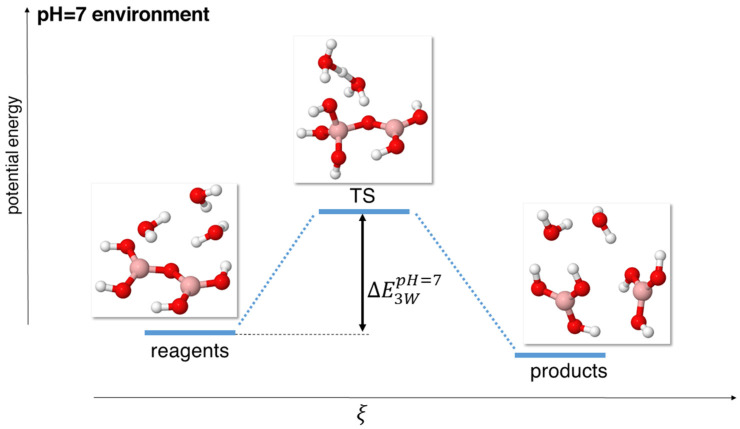
Scheme of reaction pathway for the hydrolysis mechanism at neutral pH with three water molecules. ξ represents the reaction coordinate. The energies are not to scale; see Table 2 for a complete report on the energy values.

Instead, models using three water molecules gave rise to a TS with a 7-term ring (see Figure 2), resulting in much lower transition energies (0.478 eV at the MP2 level), as shown in Table 3.

It must be pointed out that while the reaction model with just one water molecule, the DFT methods yielded reaction barriers about 0.5 eV lower than the MP2 result, in the model using three water molecules, this difference was significantly quenched. Indeed, ωB97XD approximated the MP2 result up to 0.01 eV, and B3LYP and PBE0 showed differences of about 0.1 and 0.2 eV, respectively. Also, some functional might yield an energy for products higher than that of the reactants, about hundredths of an eV.

### 2.4. Acidic pH System

In presence of an H_3_O^+^ cation, to mimic acidic conditions, the energy landscape of the reaction changes significantly. In particular, in the neutral environment, we were able to locate a single transition state, involving both the protonation of the bridging oxygen and the hydroxylation of a B atom; in acidic conditions, we observed these two processes occurring in sequence, in two different stationary points.

Namely, we consistently observed the protonation of the O_bridge_ atom in the transition state TS_1_, which was followed by an intermediate state INT where a water molecule bound to a boron atom, thus making the coordination of the latter change from trigonal to tetrahedral. This was followed by a second transition state that we indicated as TS_2_, where the bond between the bridging oxygen and the tetrahedrally coordinated boron atom was weakened and lengthened, until it eventually broke to give rise to the products.

**Figure 3:**
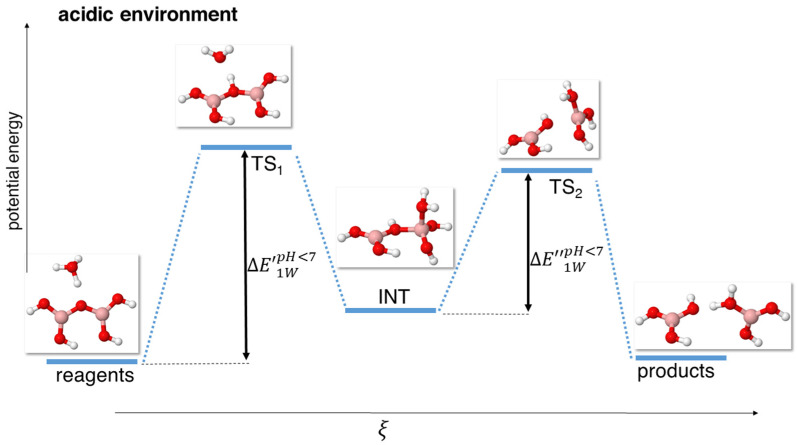
Scheme of reaction pathway for the hydrolysis mechanism at acidic pH with a single hydroxonium cation. ξ represents the reaction coordinate. The energies are not to scale; see Table 4 for a complete report on the energy values.

**Figure 4:**
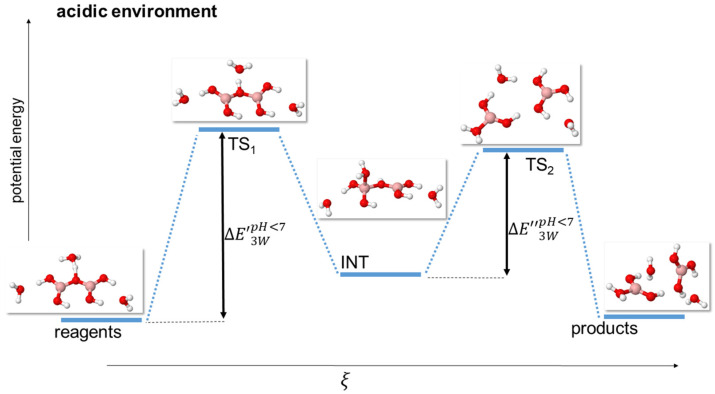
Scheme of reaction pathway for the hydrolysis mechanism at acidic pH with a single hydroxonium cation and two water molecules. ξ represents the reaction coordinate. The energies are not to scale; see Table 4 for a complete report on the energy values.

It is important to point out that we found this mechanism occurring both in the model with just one H_3_O^+^ cation (named 1 W, see Figure 3), and in that with one H_3_O^+^ cation and two water molecules (named 3 W, see Figure 4), at all levels of theory.

The presence of three stationary points between the reagents and products (two transition states and one intermediate) in the case of the acidic mechanism, instead of just one (a single, concerted transition state) as in the case of the mechanism at neutral conditions, dramatically lowers the energy barrier for the 1 W model, as reported in Table 4.

Also, it must be highlighted that for the 1 W model, the first energy barrier, $\Delta {E’}_{1W}^{pH<7}$, defined as the energy difference between the reagents and the TS_1_, was significantly higher than the second one, $\Delta {E’’}_{1W}^{pH<7}$, defined as the energy difference between the intermediate (INT) and the TS_2_. A graphical representation of both $\Delta {E’}_{1W}^{pH<7}$ and $\Delta {E’’}_{1W}^{pH<7}$ is shown in Figure 3. This means that the rate-determining step in this case is the protonation of the bridging oxygen. The actual energies found at the various levels of theory for this model are reported in Table 4.

The situation is more complicated in the 3 W model due to the increased complexity of the system. Indeed, at the DFT level of theory, the specific choice of the functional can even yield a *negative* energy for either $\Delta {E’}_{3W}^{pH<7}$ (M06HF functional) or $\Delta {E’’}_{3W}^{pH<7}$ (CAM-B3LYP, BLYP, HSE06, PBE, and M06 functionals); this occurs since the TS2 has a negative vibrational frequency, but it still had a lower energy with respect to the intermediate INT. A graphical representation of both $\Delta {E’}_{3W}^{pH<7}$ and $\Delta {E’’}_{3W}^{pH<7}$ is shown in Figure 4, while the actual energies are reported in Table 5.

We investigated the reasons behind this behavior, and found them to be dependent on these functional giving a higher hydrogen bond stabilization energy, which significantly lowered the energy of the TS_2_.

This also explains why the 1 W model, which employs just a single H_3_O^+^ cation, did not exhibit this behavior, since in that case, the number of hydrogen bonds potentially skewing the energy budget was smaller.

In fact, using these functionals to perform single-point calculations on the stationary points found and optimized at the MP2 level produced a behavior that is largely correct but, as expected, they cannot recognize transition states or intermediates based on their vibrational frequencies. We want to underline here that B3LYP, ωB97XD, and PBE0 all gave energies and energy differences close to those found at the MP2 level, with differences of less than 0.1 eV.

Overall, the energy barriers at the MP2 level and using B3LYP, ωB97XD, and PBE0 functionals yielded similar results in the 1 W and 3 W models; this can be explained by the lack of a concerted transition state in acidic conditions that (energetically) benefits from having a larger amount of water molecules explicitly included into the model. Therefore, different from the neutral simulations, in this case, the 1 W model seemed to be reliable for obtaining accurate energy barriers. However, for the functionals that poorly reproduce H-bonds, the 3 W model could indeed lead to erratic results.

### 2.5. Basic pH System

**Figure 5:**
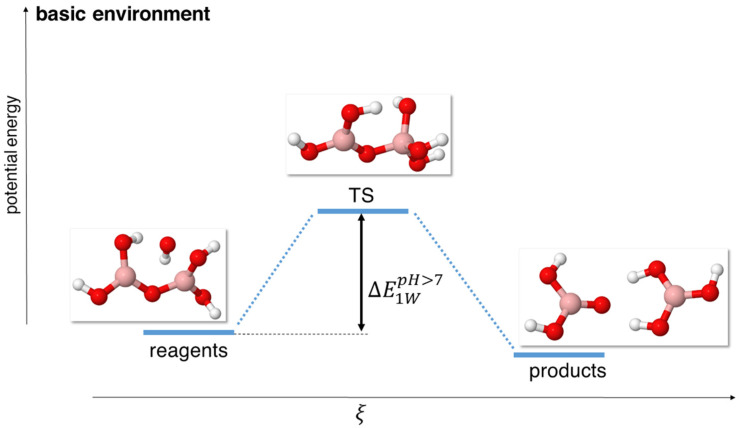
Scheme of reaction pathway for the hydrolysis mechanism at alkaline pH with a single OH^−^ anion. ξ represents the reaction coordinate. The energies are not to scale; see Table 5 for a complete report on the energy values.

**Figure 6:**
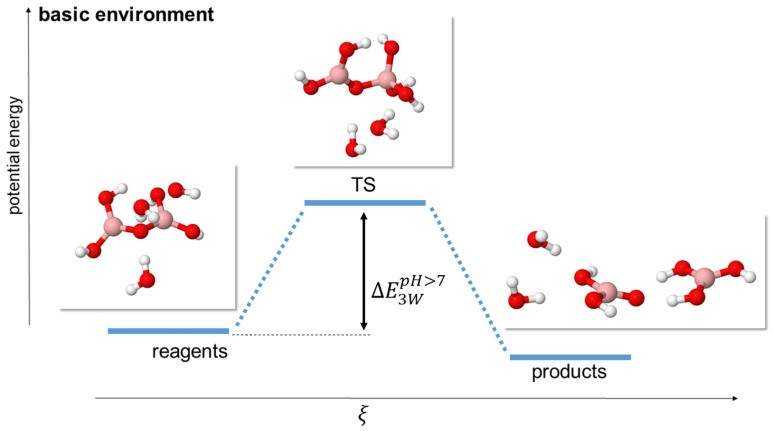
Scheme of reaction pathway for the hydrolysis mechanism at alkaline pH with a single OH^−^ anion and 2 water molecules. ξ represents the reaction coordinate. The energies are not to scale; see Table 5 for a complete report on the energy values.

We simulated alkaline conditions by using a single hydroxyl anion in our system, combined with 0 (model 1 W) or 2 (model 3 W) neutral water molecules. The hydroxyl anion attacks a boron atom, turning its coordination from trigonal to tetrahedral, giving rise to a single TS and, thus, to a rather simple energy pathway (see Figure 5 and Figure 6).

In particular, it must be noted that the reaction barrier became extremely small even for the 1 W model, on the order of about 0.1 eV at the MP2, B3LYP, PBE0, and ωB97XD levels of theory, as reported in Table 6. In fact, the reaction barrier was so small that we had issues optimizing the reagents, as they very easily evolved into the transition state, which was very close in energy. Also, the reaction evolved without the need for the protonation of the bridging oxygen, which is different from the mechanisms in the neutral and acidic conditions.

In the more complex 3 W model, we observed that the reaction barriers decreased (see Table 7) while maintaining a rather similar transition geometry for the B_2_O_5_H_4_ and OH^−^ fragments but with a ring-like disposition for the other two additional water molecules. This decrease in the reaction barriers occurred at all levels of theory.

## 3. Computational Details

All QM calculations of the molecule–metal complexes presented here were performed with the Gaussian 16 suite of programs [23]. To produce the reference reaction energy profiles, we resorted to the 2nd order Moeller–Plesset perturbation theory approach (MP2) [24] and CCSD(T) calculations using 6-311++G(d,p) and Apr-cc-pVTZ basis sets. The final choice of MP2 as the reference for the ab initio method is discussed in more detail in Section 2.2.

Regarding density functional theory calculations, a plethora of exchange–correlation functionals were used, namely hybrid B3LYP [25,26], PBE0 [27], M06 [28], M06HF [29], HSE06 [30], CAM-B3LYP [31], ωB97Xd [32], and GGA and meta-GGA like BLYP [26,33], PBE [34], TPSS [35]. B3LYP, PBE0, M06, and M06HF, which are general hybrid functionals with different percentages of Hartree–Fock exchange (for instance, 100% for M06HF, 25% for PBE0). HSE06, CAM-B3LYP, and ωB97Xd are instead range-separated hybrids, the first is based on PBE, the second on B3LYP, and the third one also includes dispersion effects. BLYP, PBE, and TPSS are “pure” functionals, and do not use any Hartree–Fock exchange.

All the DFT calculations adopted Pople’s all-electrons 6-311++G(d,p) basis set, in combination with the Grimme’s GD3BJ correction [36] or the simpler GD3 correction [37] of dispersion interactions (when the former is not available for a specific DFT functional). For the ωB97Xd functional, no further correction was adopted.

In all calculations, the linear-response polarizable continuum model (PCM) [38,39] was adopted to include bulk solvent effects.

### Search and Assessment of Transition States 

The discovery of the transition states is, notoriously, a rather subtle research approach, and for this reason, we carried it out with 3 possible approaches: (1) performing optimizations with the Berny algorithm (corresponding to the TS keyword in Gaussian 16); (2) using the QST2/QST3 approach, thus giving a guess of the starting reagents and final product (and, in case of QST3, of the transition state as well) and letting the Transit-Guided Quasi-Newton algorithm optimize the transition state; and (3) stretching what we supposed was the reaction coordinates, performing a relaxed scan on it, and looking for a maximum of the potential energy.

Notwithstanding the procedure we adopted to find the transition states, these latter have been validated in two ways: (a) by the presence of a single imaginary frequency in the Hessian and (b) by using the intrinsic reaction coordinate approach (IRC keyword in Gaussian) to check that following the forward and reverse reaction pathways lead to the products and the reactants, respectively.

## 4. Concluding Remarks

In this study, we performed a comprehensive ab initio and Density Functional Theory (DFT) investigation of the hydrolysis mechanism and energetics of a borate network. Our focus was on modeling a system comprising two boron (B) atoms and oxygen (O) bridging atoms, along with various water molecules, to examine the interaction dynamics where water attacks and breaks the network. Additionally, we incorporated either hydroxide (OH^−^) or proton (H^+^) ions to simulate basic or acidic environments, respectively.

The motivation for this research stems from the challenges in obtaining reliable experimental data regarding the hydrolysis pathway and energies. These energetic insights are crucial for subsequent studies, both in computational realms, such as machine learning models that require accurate reaction energies for calibration, and in experimental setups seeking a reliable reference point.

Our methodology included both ab initio calculations at the MP2 level of theory and DFT employing many different functionals.

The results obtained for neutral conditions exhibited reaction barriers greater than 1 eV, which is qualitatively of the same order of magnitude as the existing ab initio results for silica networks [13].

The principal findings of our research indicate that the borate network is significantly more susceptible to hydrolysis in a basic environment, followed by neutral and acidic environment. Indeed, we observed a drop in the activation energy from about 0.5 eV (neutral or acidic environment) to less than 0.1 eV (alkaline environment). These data are significantly lower than those found for silicate structures where the hydrolysis reaction barriers exceeded 1 eV [13].

Furthermore, our study reveals that the inclusion of a greater number of water molecules explicitly into the calculations significantly influences the results when the transition state has a concerted, ring-like topology. Specifically, it reduces the energy of the reaction barriers, with the transition states predominantly forming closed rings composed of water and the B-O-B network. This structural preference in transition geometries demonstrates why including additional explicit water molecules in the calculations results in lower reaction energies.

In terms of accuracy, we observed that for the hydrolysis reactions studied here, the B3LYP, PBE0, and ωB97Xd functionals exhibited an overall better performance, i.e., these functionals qualitatively reproduced the MP2-calculated reaction paths and quantitatively matched the reaction barriers within an approximate range of 0.1–0.2 eV for the most reasonable pathways. CAM-B3LYP also gave a similar accuracy with respect to the MP2 data [19] except for the most complicated reaction pathway involving two transition states and an intermediate.

Still, we must point out that, currently, no approximated functional [40] is known to be able to correctly and dependably reproduce the reaction barriers in chemical reactions. In fact, the description of reaction barriers is problematic for (1) GGA-type functionals because they might underestimate reaction barriers up to the eV scale (due to the very local nature of their functional form), while (2) non-local functionals involving Hartree–Fock exchange like general and range-separated hybrids might also lead to wrong results since they exhibit a spurious long-range repulsive behavior. We have also to point out Hait’s and Head-Gordon’s words: “*it is difficult for a single functional to be effective at addressing delocalization error over many species*” [41]. This is particularly an issue as boron oxides are not usually included in most libraries on which density functional approximations are tested.

However, in ref. [41], it was observed that functionals belonging to the CAM-B3LYP, ωB97, and PBE0 families showed surprising good behaviors in dealing with delocalization errors in the studied systems with fractional charges. This was also observed in ref. [42]. 

Also, part of the delocalization issue can be mitigated using dispersion corrections (as we did here) that, at least, should lead to more reliable relaxed structures as they partially account for some of the long-range forces.

While the subject of assessing DFT methods for hydrolysis reactions is a continuously researched subject (see for instance ref. [20]), here, we wanted to point out that we adopted an approach consisting of optimizing the structures at all levels of theory, as some of us already did previously [43,44]; this has the drawback that small changes in the geometry of the optimized structure can significantly change the energies of the models, contributing to the scattered nature of the results and somewhat obscuring the many contributions to the energies [41].

However, the great advantage of this approach is that it allows future investigators to directly apply the findings of this study that also include the structural relaxation, increasing the usefulness of this paper.

Indeed, this study provides valuable insights into the hydrolysis mechanism of borate networks, contributing to a deeper understanding of the process and offering a reliable computational reference for future experimental and computational investigations. The findings underscore the importance of environmental factors, like pH conditions, the composition of the reacting system, particularly the role of water molecules, in determining the hydrolysis behavior of borate networks, and the importance of the topology of the transition state(s). The agreement of our DFT results with the MP2 calculations reinforces the potential of DFT methods in accurately modeling complex chemical reactions, particularly in cases where the experimental data are scarce or otherwise challenging to obtain.

## Figures and Tables

**Table 2:** **DFT and MP2** Energies for the stationary points of the reaction pathway at neutral pH with 1 water molecule. The energies have been rescaled to set the energy of the reagents to zero. The energy of the TS coincides with $\Delta E_{1W}^{pH=7}$.

Level of Theory	Stationary Point	Energy/eV
MP2	Reagents	0
	TS	2.030
	Product	−0.015
B3LYP	Reagents	0
	TS	1.492
	Products	−0.046
CAM-B3LYP	Reagents	0
	TS	1.403
	Products	−0.052
BLYP	Reagents	0
	TS	1.457
	Products	0.050
PBE0	Reagents	0
	TS	1.338
	Products	−0.046
HSE06	Reagents	0
	TS	1.327
	Products	−0.077
PBE	Reagents	0
	TS	1.200
	Products	−0.054
TPSS	Reagents	0
	TS	1.233
	Products	−0.040
M06HF	Reagents	0
	TS	1.078
	Products	−0.005
M06	Reagents	0
	TS	1.508
	Products	−0.022
ωB97XD	Reagents	0
	TS	1.418
	Products	−0.042

**Table 3:** **DFT and MP2** Energies for the stationary points of the reaction pathway at neutral pH with 3 water molecules. The energies have been rescaled to set the energy of the reagents to zero. The energy of the TS coincides with $\Delta E_{3W}^{pH=7}$.

Level of Theory	Stationary Point	Energy/eV
MP2	Reagents	0
	TS	0.478
	Product	−0.189
B3LYP	Reagents	0
	TS	0.572
	Products	−0.146
CAM-B3LYP	Reagents	0
	TS	0.395
	Products	−0.211
BLYP	Reagents	0
	TS	0.393
	Products	−0.347
PBE0	Reagents	0
	TS	0.363
	Products	−0.148
HSE06	Reagents	0
	TS	0.112
	Products	−0.210
PBE	Reagents	0
	TS	0.252
	Products	−0.196
TPSS	Reagents	0
	TS	0.303
	Products	−0.132
M06HF	Reagents	0
	TS	0.178
	Products	0.004
M06	Reagents	0
	TS	0.038
	Products	−0.236
ωB97XD	Reagents	0
	TS	0.482
	Products	−0.213

**Table 4:** **DFT and MP2** Energies for the stationary points of the reaction pathway at acidic pH with 1 H_3_O^+^ cation. The energies have been rescaled to set the energy of the reagents to zero. The $\Delta {E’}_{1W}^{pH<7}$ and $\Delta {E’’}_{1W}^{pH<7}$ energy differences are reported separately in a different column.

Level of Theory	Stationary Point	Energy/eV	$\Delta {E’}_{1W}^{pH<7}$/eV	$\Delta {E’’}_{1W}^{pH<7}$/eV
MP2	Reagents	0		
	TS1	0.467	0.467	
	INT	0.384		
	TS2	0.401		0.018
	Products	0.003		
B3LYP	Reagents	0		
	TS1	0.623	0.623	
	INT	0.445		
	TS2	0.496		0.050
	Products	0.080		
CAM-B3LYP	Reagents	0		
	TS1	0.618	0.618	
	INT	0.417		
	TS2	0.566		0.128
	Products	−0.051		
BLYP	Reagents	0		
	TS1	0.643	0.618	
	INT	0.541		
	TS2	0.590		0.048
	Products	−0.079		
PBE0	Reagents	0		
	TS1	0.608	0.608	
	INT	0.378		
	TS2	0.554		0.177
	Products	−0.049		
HSE06	Reagents	0		
	TS1	0.489	0.489	
	INT	0.421		
	TS2	0.564		0.143
	Products	−0.059		
PBE	Reagents	0		
	TS1	0.719	0.719	
	INT	0.462		
	TS2	0.516		0.055
	Products	−0.057		
TPSS	Reagents	0		
	TS1	0.504	0.504	
	INT	0.402		
	TS2	0.566		0.164
	Products	−0.043		
M06HF	Reagents	0		
	TS1	0.307	0.307	
	INT	0.225		
	TS2	0.587		0.363
	Products	0.045		
M06	Reagents	0		
	TS1	0.594	0.594	
	INT	0.413		
	TS2	0.528		0.115
	Products	−0.046		
ωB97XD	Reagents	0		
	TS1	0.637	0.637	
	INT	0.396		
	TS2	0.496		0.099
	Products	−0.062		

**Table 5:** **DFT and MP2** Energies for the stationary points of the reaction pathway at acidic pH with 1 H_3_O^+^ cation and 2 water molecules. The energies have been rescaled to set the energy of the reagents to zero. The $\Delta {E’}_{3W}^{pH<7}$ and $\Delta {E’’}_{3W}^{pH<7}$ energy differences are reported separately in a different column.

Level of Theory	Stationary Point	Energy/eV	$\Delta {E’}_{3W}^{pH<7}$/eV	$\Delta {E’’}_{3W}^{pH<7}$/eV
MP2	Reagents	0		
	TS1	0.530	0.530	
	INT	0.448		
	TS2	0.459		0.011
	Products	0.095		
B3LYP	Reagents	0		
	TS1	0.568	0.568	
	INT	0.556		
	TS2	0.610		0.054
	Products	0.364		
CAM-B3LYP	Reagents	0		
	TS1	0.236	0.236	
	INT	0.745		
	TS2	0.043		−0.695
	Products	0.591		
BLYP	Reagents	0		
	TS1	0.582	0.582	
	INT	0.527		
	TS2	−0.252		−0.778
	Products	0.373		
PBE0	Reagents	0		
	TS1	0.549	0.549	
	INT	0.721		
	TS2	0.825		0.104
	Products	0.314		
HSE06	Reagents	0		
	TS1	0.867	0.867	
	INT	0.765		
	TS2	0.227		−0.538
	Products	0.465		
PBE	Reagents	0		
	TS1	0.836	0.836	
	INT	0.801		
	TS2	0.209		−0.591
	Products	0.624		
TPSS	Reagents	0		
	TS1	0.668	0.668	
	INT	0.740		
	TS2	0.211		−0.529
	Products	0.617		
M06HF	Reagents	0		
	TS1	−0.100	−0.100	
	INT	0.266		
	TS2	0.310		0.043
	Products	0.323		
M06	Reagents	0		
	TS1	0.112	0.112	
	INT	0.491		
	TS2	0.020		−0.462
	Products	0.325		
ωB97XD	Reagents	0		
	TS1	0.549	0.549	
	INT	0.484		
	TS2	0.498		0.014
	Products	0.322		

**Table 6:** **DFT and MP2** Energies for the stationary points of the reaction pathway at basic pH with 1 OH^−^. The energies have been rescaled to set the energy of the reagents to zero. The energy of the TS coincides with $\Delta E_{1W}^{pH>7}$.

Level of Theory	Stationary Point	Energy/eV
MP2	Reagents	0
	TS	0.098
	Product	−0.002
B3LYP	Reagents	0
	TS	0.126
	Products	−0.062
CAM-B3LYP	Reagents	0
	TS	0.131
	Products	−0.073
BLYP	Reagents	0
	TS	0.270
	Products	−0.069
PBE0	Reagents	0
	TS	0.122
	Products	−0.036
HSE06	Reagents	0
	TS	0.155
	Products	0.011
PBE	Reagents	0
	TS	0.371
	Products	−0.041
TPSS	Reagents	0
	TS	0.339
	Products	−0.140
M06HF	Reagents	0
	TS	0.137
	Products	−0.672
M06	Reagents	0
	TS	0.288
	Products	−0.275
ωB97XD	Reagents	0
	TS	0.087
	Products	−0.042

**Table 7:** **DFT and MP2** Energies for the stationary points of the reaction pathway at basic pH with 1 OH^−^ and 2 water molecules. The energies have been rescaled to set the energy of the reagents to zero. The energy of the TS coincides with $\Delta E_{3W}^{pH>7}$.

Level of Theory	Stationary Point	Energy/eV
MP2	Reagents	0
	TS	0.062
	Product	−0.013
B3LYP	Reagents	0
	TS	0.080
	Products	−0.068
CAM-B3LYP	Reagents	0
	TS	0.089
	Products	−0.083
BLYP	Reagents	0
	TS	0.251
	Products	−0.071
PBE0	Reagents	0
	TS	0.083
	Products	−0.044
HSE06	Reagents	0
	TS	0.094
	Products	0.009
PBE	Reagents	0
	TS	0.239
	Products	−0.057
TPSS	Reagents	0
	TS	0.247
	Products	−0.148
M06HF	Reagents	0
	TS	0.099
	Products	−0.566
M06	Reagents	0
	TS	0.165
	Products	−0.234
ωB97XD	Reagents	0
	TS	0.076
	Products	−0.038

## Data Availability

Data are contained within the article.
